# Correlation Between Thioredoxin-Interacting Protein and Nerve Conduction Velocity in Patients With Type 2 Diabetes Mellitus

**DOI:** 10.3389/fneur.2020.00733

**Published:** 2020-07-22

**Authors:** Yuan Gao, Shuchun Chen, Minmin Peng, Zi Wang, Luping Ren, Shumin Mu, Meiling Zheng

**Affiliations:** ^1^Department of Endocrinology, Hebei General Hospital, Shijiazhuang, China; ^2^Graduate School of North China University of Science and Technology, Tangshan, China; ^3^Graduate School of Hebei North University, Zhangjiakou, China; ^4^Graduate School of Hebei Medical University, Shijiazhuang, China

**Keywords:** type 2 diabetes mellitus, TXNIP, peripheral nerve conduction velocity, oxidative stress, tumor necrosis factor alpha

## Abstract

**Aims:** To investigate the correlation between thioredoxin-interacting protein (TXNIP) and peripheral nerve conduction velocity (NCV) in patients with type 2 diabetes mellitus.

**Methods:** In total, 338 patients with type 2 diabetes mellitus (T2DM) were included in this study. We collected the clinical data and measured the motor conduction velocities of the bilateral ulnar nerve, median nerve, tibial nerve, and common peroneal nerve, and the sensory conduction velocities of the ulnar nerve, median nerve, sural nerve, and superficial peroneal nerve. According to the results, the patients were divided into two groups: normal peripheral nerve conduction group (NCVN group) and abnormal peripheral nerve conduction group (NCVA group). The two groups were then compared in terms of the conventional biochemical index and the sugar metabolic index as well as the serum levels of TXNIP, reduced glutathione (GSH), total superoxide dismutase (SOD), malondialdehyde (MDA), and tumor necrosis factor alpha (TNF-α). The correlation between TXNIP and NCV was also analyzed.

**Results:** Compared with the NCVN group, the TXNIP and MDA values were significantly increased in the NCVA group (*P* < 0.05). Among the patients with T2DM, age, fasting glucose, SDBG, and TXNIP were risk factors for NCV abnormality, while vitamin D3 was a protective factor. After adjusting for related confounding factors, TXNIP was significantly correlated with NCV (*P* < 0.05). Among the patients with T2DM, TXNIP was an independent risk factor for left ulnar motor conduction velocity (MCV), right ulnar MCV, left median MCV, and right median MCV. TNF-α was identified as a positive influencing factor for serum TXNIP, while serum TXNIP was a positive factor for TNF-α and MDA (both *P* < 0.05).

**Conclusion:** Serum TXNIP is related to NCV in T2DM patients. In combination with oxidative stress and inflammation, TXNIP may affect diabetic peripheral neuropathy (DPN).

## Introduction

Diabetic peripheral neuropathy (DPN) is a common complication of diabetes mellitus (DM), and occurs in 30–60% of diabetic patients (1). The pathogenesis of DPN includes metabolic abnormality, insulin resistance, growth factor deficiency, oxidative stress, and inflammatory factor activation. Among these factors, oxidative stress plays an important role in peripheral neuropathy. Hyperglycemia causes oxidative stress, activates proinflammatory prealbumin, and damages mitochondrial function through multiple pathways, resulting in impaired downstream transmission of nerve impulses, loss of neurotrophic support, which eventually lead to the production of DPN (2).

TXNIP was first identified in HL-60 promyelocytic leukemia cells treated with 1,25-dihydroxyvitamin D3 (3). It is a 46-kD protein composed of 391 amino acid residues that is encoded on human chromosome 1q21.1 and is expressed in many tissues (4). TXNIP is an endogenous inhibitor of thioredoxin (TRX), also known as vitamin D3 upregulating protein-1 (VDUP-1) or thioredoxin-binding protein-2 (TBP-2). TXNIP inhibits TRX expression and function. TXNIP-mediated oxidative stress plays an important role in the occurrence and development of diabetic nephropathy, diabetic retinopathy, and arteriosclerosis (5). Studies have shown that TXNIP can affect the development of diabetic nephropathy by inhibiting endogenous oxidative stress (6). Zhao et al. found a correlation between TXNIP and carotid intima media thickness (CIMT) (7). TXNIP also affects type 2 diabetic retinopathy by inducing retinal inflammation and enhancing oxidative stress (8). Animal studies have shown that TXNIP expression is increased in the dorsal root ganglia of diabetic rats, and oxidative stress in DPN decreases with decreasing TXNIP expression (9). To date, no clinical studies on serum TXNIP and DPN in T2DM patients have been reported. NCV is considered to be the most sensitive, accurate, and reliable marker of DPN (10). In this study, we investigated the relationship between serum TXNIP levels and NCV in patients with type 2 diabetes to provide a new target for the prevention and treatment of DPN.

## Subjects and Methods

### Subjects

A total of 338 T2DM patients who were treated in Hebei General Hospital from December 2018 to August 2019 were selected. T2DM was diagnosed in accordance with the 1999 WHO diagnostic criteria for diabetes: diabetes symptoms plus plasma glucose at any time ≥11.1 mmol/L, or fasting blood glucose (FBG) ≥7.0 mmol/L, or 2-h oral glucose tolerance test (OGTT) glucose ≥11.1 mmol/L. The patients were divided into two groups according to the nerve conduction velocity (NCV) (Keypoint Myoelectric Evoked Potentiometer [Alpine Biomed ApS; Dantec, Denmark] normal value, the specific normal value is shown in the Appendix): normal nerve conduction group (NCVN; 154 cases, 106 male and 48 female; average age 52.69 ± 11.45 years) and abnormal nerve conduction group (NCVA; 184 cases, 117 male and 67 female; average age 58.90 ± 12.20 years).

The following exclusion criteria were applied: age >80 or <20 years; acute complications of diabetes; severe disease of the heart, liver, kidney, or other important organs; acute or chronic infections, autoimmune diseases, hematological diseases; vitamin deficiencies; malignant tumors (no abnormality in tumor-related laboratory indexes); thyroid dysfunction; numbness of the limbs caused by cervical spondylosis, lumbar disc herniation, or other reasons; patients with alcohol-addiction or taking hormones and patients with family history of neuropathy. Patients and/or family members provided written informed consent. This study was approved by the Clinical Research Ethics Committee of Hebei General Hospital (China).

### Methods

#### Collection of Basic Information

For each participant, the basic information (name, age, duration of diabetes, medication, and other general conditions) was collected and recorded in an Excel spreadsheet. The measurement of the basic patient parameters and the seven-point blood glucose measurements were performed by the same group of nurses. The systolic blood pressure (SBP), diastolic blood pressure (DBP), height, and weight on the day of admission were recorded, and used to calculate the following: body mass index (BMI) [BMI = weight (kg)/height (m)^2^], insulin resistance index (HOMA-IR) [FBG (mmol/L) × fasting insulin (mIU/L)/22.5]; mean post-prandial glucose excursion (PPGE) [the absolute value of the difference between the 2-h blood glucose concentration after three meals and the corresponding pre-meal glucose concentration]; standard deviation of blood glucose (SDBG) at 7 a.m.; largest amplitude of glucose excursion (LAGE): difference between maximum and minimum blood glucose values in 1 day; mean blood glucose (MBG) at 7 a.m.

#### Laboratory Data Collection

Fasting venous blood was collected from patients who fasted for more than 8 h prior to testing for serum albumin (Alb), alanine aminotransferase (ALT), aspartate aminotransferase (AST), triglycerides (TG), total cholesterol (TC), high density lipoprotein cholesterol (HDL-C), low density lipoprotein cholesterol (LDL-C), creatinine (Cr), blood urea nitrogen (BUN), glomerular filtration rate (GFR), uric acid (UA), fasting blood glucose (FBG); these indicators were analyzed by professionals using a fully automatic biochemical analyzer. Glycated hemoglobin (HbAlc), total triiodothyronine (TT3), total thyroxine (TT4), thyroid stimulating hormone (TSH), and 25-hydroxyvitamin D3 were all determined by the laboratory physician using an electrochemical luminescence method. Superoxide dismutase (SOD), malondialdehyde (MDA), and glutathione (GSH) were measured by professionals to using colorimetric tests (Wuhan Elite Co., Ltd.). Serum TXNIP and tumor necrosis factor-α (TNF-α) expression levels were measured using enzyme-linked immunosorbent assay (ELISA) kits (Wuhan Elite Corporation, China) according to the manufacturer's instructions.

#### The Measurement of NCV

NCV was recorded using the Keypoint Myoelectric Evoked Potentiometer (Alpine Biomed ApS; Dantec, Denmark) under standard surface stimulation. During the measurement, the electrode was coated with conductive glue and fixed with tape. NCV was measured on both sides of the limbs. The tests were conducted at room temperature (20–25°C), with a stimulation frequency of 1 Hz, a stimulation pulse width of 0.1 ms, and sensitivity of 5 mV/lattice. The skin temperature of the patients was measured using an infrared thermal imager and controlled between 33 and 34°C. For the investigation of motor nerves, we selected the median, ulnar, tibial, and common peroneal nerves. For the investigation of sensory nerves, we selected the median, ulnar, sural, and superficial peroneal nerves.

## Statistical Analysis

All data were analyzed using SPSS 21.0 software. The measurement data that conformed to the normal distribution were expressed as the mean ± standard deviation (*SD*). The measurement data that did not conform to the normal distribution were expressed as the median (interquartile range). For comparisons of the two groups of data, *t*-tests of independent samples were used for the normal distribution data, and Mann–Whitney *U*-tests were used for skewed distribution data. Chi-squared tests were used to compare the categorical variables between the two groups. Multivariate linear and logistic regression analysis was used to analyze the influencing factors. *P* < 0.05 was considered to indicate statistical significance.

## Results

### Comparison of Indexes Between NCVN Group and NCVA Group

**Figure 1:**
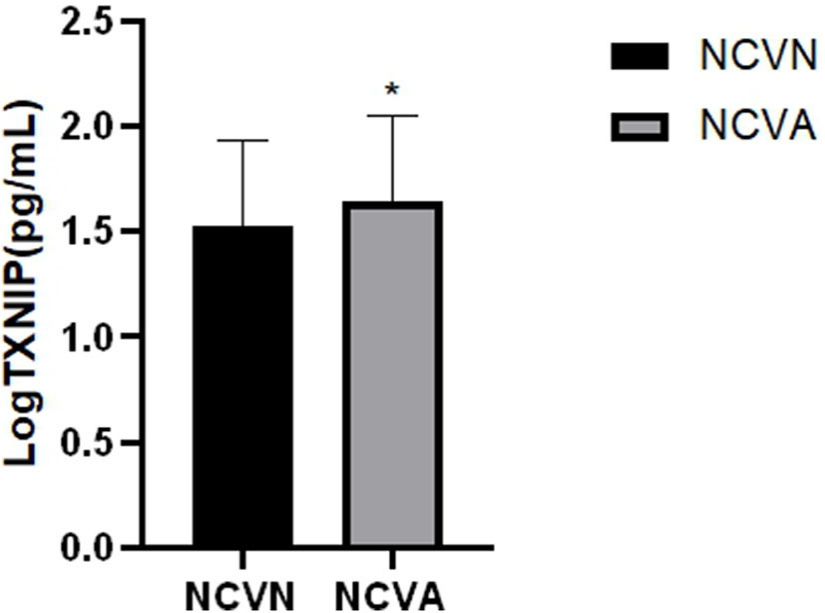
Serum TXNIP levels in the NCVN and NCVA groups. TXNIP level was evaluated using ELISA, Values represent the means ± SEM in the NCVN and NCVA groups. *P < 0.05: NCVA vs. NCVN.

There were no significant differences in sex, BMI, HOMA-IR, SBP, TG, TC, HDL, LDL, AST, Cr, UA, TT3, TT4, TSH, PPGE, GSH, TNF-α, and SOD between the NCVN group and NCVA group (*P* > 0.05). Compared with the NCVN group, the age, course of diabetes, use of insulin, use of metformin, FBG, HbA1c, BUN, SDBG, LAGE, MBG, MDA, and TXNIP values significantly higher in the NCVA group (Figure 1) (*P* < 0.05). Compared with the NCVN group, the DBP, ALB, ALT, GFR, and vitamin D3 were significantly lower in the NCVA group (*P* < 0.05, Table 1) (It should be noted that because the TXNIP data did not conform to the normal distribution, it was logarithmically transformed before analysis to improve the normality).

**Table 1:** Comparison of indicators between the NCVN and NCVA groups.

	**NCVN**	**NCVA**	***P***
*n*	154	184	
Sex (M/F)	106/48	117/67	0.311
Age (years)	52.69 ± 11.45	58.90 ± 12.20	0.000^**^
Diabetes course (years)	6.00 (2.00, 11.00)	9.50 (3.00, 15.75)	0.005^**^
BMI (kg/m^2^)	25.83 ± 3.73	26.57 ± 3.78	0.075
SBP (mmHg)	132.47 ± 20.75	135.59 ± 20.49	0.167
DBP (mmHg)	83.23 ± 12.64	80.93 ± 14.13	0.119
Alb (g/L)	42.07 ± 2.97	40.13 ± 3.71	0.000^**^
ALT (U/L)	19.80 (14.80, 28.20)	17.15 (11.92, 25.30)	0.011^*^
AST (U/L)	19.50 (16.40, 23.00)	19.10 (15.15, 25.35)	0.759
Cr (μmol/L)	71.10 (63.60, 80.30)	73.3 (64.74, 83.58)	0.081
BUN (mmol/L)	5.10 (4.47, 6.00)	5.61 (4.50, 6.80)	0.030^*^
UA (μmol/L)	308.18 ± 83.79	314.47 ± 92.40	0.525
GFR (ml/min)	97.52 (90.52, 106.89)	90.91 (76.53, 102.48)	0.000^**^
TC (mmol/L)	4.80 ± 1.14	4.79 ± 1.56	0.929
TG (mmol/L)	1.49 (0.99, 2.35)	1.43 (1.07, 2.12)	0.993
HDL-C (mmol/L)	1.11 (0.91, 1.47)	1.13 (0.91, 1.68)	0.838
LDL-C (mmol/L)	2.68 ± 1.06	2.65 ± 1.24	0.822
FBG (mmol/L)	7.75 (6.36, 9.90)	8.79 (6.67, 11.62)	0.005^**^
HOMA-IR	2.78 (1.58, 5.01)	3.36 (1.87, 6.67)	0.130
HbA1c (%)	8.25 ± 1.94	9.30 ± 1.95	0.000^**^
PPGE (mmol/L)	3.38 ± 1.79	3.49 ± 1.63	0.559
SDBG (mmol/L)	2.13 ± 1.07	2.55 ± 1.04	0.001^**^
LAGE (mmol/L)	6.25 ± 3.04	7.25 ± 3.02	0.004^**^
MBG (mmol/L)	9.68 ± 2.21	10.43 ± 2.44	0.006^*^
TT3 (nmol/L)	1.49 (1.32, 1.66)	1.41 (1.30, 1.63)	0.155
TT4 (nmol/L)	89.50 (78.92, 101.85)	90.31 (78.97, 100.85)	0.818
TSH (μIU/mL)	1.99 (1.12, 2.86)	2.02 (1.40, 2.83)	0.373
25(OH)D3 (ng/mL)	16.79 (13.88, 21.39)	15.43 (12.35, 19.65)	0.026^*^
SOD (μmol/L)	59.48 ± 8.91	60.25 ± 9.31	0.442
GSH (μmol/L)	19.46 (10.95, 44.47)	18.85 (12.13, 36.67)	0.744
MDA (μmol/L)	13.47 (8.09, 21.95)	17.81 (9.84, 23.57)	0.030^*^
TNF-α (μmol/L)	28.24 (18.29, 42.50)	34.16 (19.93, 48.12)	0.117
LogTXNIP (pg/mL)	1.53 ± 0.40	1.64 ± 0.41	0.018^*^
Use of insulin (Y/N)	81/73	128/56	0.001^**^
Use of metformin (Y/N)	103/51	91/93	0.001^**^

* *P < 0.05*,

** *P < 0.01 NCVA vs. NCVN*.

*BMI, Body mass index; SBP, Systolic blood pressure; DBP, Diastolic blood pressure; Alb, Serum albumin; ALT, Alanine aminotransferase; AST, Aspartate aminotransferase; TG, Triglycerides; TC, Total cholesterol; HDL-C, High density lipoprotein cholesterol; LDL-C, Low density lipoprotein cholesterol; Cr, Creatinine; BUN, Blood urea nitrogen; GFR, Glomerular filtration rate; UA, Uric acid; FBG, Fasting blood glucose; HbAlc, Glycated hemoglobin; HOMA-IR, Insulin resistance index; TT3, Total triiodothyronine; TT4, Total thyroxine; TSH, Thyroid stimulating hormone; PPGE, Mean post-prandial glucose excursion; SDBG, Standard deviation of blood glucose; LAGE, Largest amplitude of glucose excursion; MBG, Mean blood glucose; SOD, Superoxide dismutase; MDA, Malondialdehyde; GSH, Glutathione; TNF-α, Tumor necrosis factor alpha*.

### Correlation Between NCV and TXNIP in T2DM Patients

Using the existence of NCV abnormality as the dependent variable, logistic regression analysis showed that age, FBG, SDBG, and TXNIP were risk factors for NCV abnormality in T2DM patients, while vitamin D3 was a protective factor for NCV abnormality (Table 2). After adjusting for age, course of diabetes, FBG, HblAc, vitamin D3, SDBG, and MBG, TXNIP was still an independent risk factor for NCV abnormality (OR = 2.001, *P* = 0.047) (Table 3).

**Table 2:** Logistic regression of risk factors for abnormal peripheral nerve conduction velocity in T2DM patients.

**Risk factors**	***B***	**S.E**.	**Wals**	**OR**	***P***	**95%CI**	
						**Lower limit**	**Upper limit**
Age	0.059	0.013	19.211	1.061	0.000	1.033	1.089
FBG	0.135	0.053	6.344	1.144	0.012	1.030	1.270
SDBG	0.311	0.157	3.905	1.365	0.048	1.003	1.858
Vitamin D3	−0.056	0.024	5.612	0.945	0.018	0.902	0.990
TXNIP	0.694	0.350	3.934	2.001	0.047	1.008	3.923

*FBG, fasting blood glucose; SDBG, standard deviation of blood glucose; TXNIP, thioredoxin-interacting protein*.

**Table 3:** Correlation between TXNIP and abnormal peripheral nerve conduction velocity.

	***B***	**S.E**.	**Wals**	**Adjusted *R^**2**^***	**95%CI**	***P***
Model 1	0.640	0.273	7.577	0.022	(1.109, 3.240)	0.019
Model 2	0.728	0.302	5.807	0.229	(1.146, 3.744)	0.016
Model 3	0.694	0.350	3.934	0.273	(1.008, 3.973)	0.047

*Model 1 is unadjusted*.

*Model 2 is adjusted for age, course of diabetes, FBG, and HblAc*.

*Model 3 is further adjusted for age, course of diabetes, FBG, HblAc, vitamin D3, SDBG, and MBG*.

### The Influence of TXNIP on NCV in T2DM Patients

To explore the influence of TXNIP on the conduction velocity of different peripheral nerves, we used the motor and/or sensory conduction velocity of the bilateral ulnar nerve, median nerve, common peroneal nerve, tibial nerve, sural nerve, and superficial peroneal nerve as dependent variables, while TXNIP, age, course of diabetes, glycosylated hemoglobin, FBG, vitamin D3, SDBG were used as independent variables. By adjusting for confounding factors (Table 4), TXNIP was identified as an independent risk factor for MCV in the left ulnar, right ulnar, left median, and right median nerves. For every unit of increase in serum LogTXNIP, left ulnar MCV decreased by 0.154 m/s, right ulnar MCV decreased by 0.131 m/s, left median MCV decreased by 0.135 m/s, and right median MCV decreased by 0.135 m/s (*P* < 0.05, Table 4).

**Table 4:** Correlation of TXNIP with different nerve conduction velocities in patients with type 2 diabetes.

	***B***	**S.E**.	**β**	**Adjusted *R*^**2**^**	***F***	**95%CI**	***P***
Left ulnar MCV	−2.092	0.790	−0.154	0.098	5.175	(−3.649, −0.536)	0.009^**^
Right ulnar MCV	−1.727	0.757	−0.131	0.122	6.316	(−3.217, −0.238)	0.023^*^
Left median MCV	−1.530	0.633	−0.135	0.174	9.085	(−2.776, −0.284)	0.016^*^
Right median MCV	−1.509	0.635	−0.135	0.142	7.318	(−2.758, −0.259)	0.018^*^
Left common peroneal MCV	−1.174	0.649	−0.105	0.113	5.786	(−2.453, 0.105)	0.072
Right common peroneal MCV	−1.112	0.637	−0.101	0.123	6.288	(−2.367, 0.142)	0.082
Left tibial MCV	−1.148	0.604	−0.108	0.167	8.540	(−2.338,0.040)	0.058
Right tibial MCV	−0.959	0.599	−0.089	0.193	9.984	(−2.139,0.221)	0.111
Left ulnar SCV	−0.598	0.856	−0.041	0.112	5.643	(−2.284, 1.087)	0.485
Right ulnar SCV	−0.622	0.864	−0.043	0.080	4.226	(−2.323, 1.080)	0.472
Left median SCV	−1.989	1.011	−0.114	0.137	6.834	(−3.981, 0.002)	0.050
Right median SCV	0.176	1.104	0.009	0.154	7.542	(−1.998, 2.350)	0.873
Left superficial peroneal SCV	−1.629	0.883	−0.116	0.076	3.827	(−3.368, 0.111)	0.066
Right superficial peroneal SCV	−0.822	1.006	−0.053	0.027	1.958	(−2.805, 1.160)	0.415
Left sural SCV	−0.352	0.928	−0.024	0.050	2.902	(−2.179,1.475)	0.705
Right sural SCV	−1.460	0.927	−0.099	0.042	2.542	(−3.287,0.366)	0.116

* *P < 0.05*,

** *P < 0.01 NCVA vs. NCVN*.

*MCV, motor nerve conduction velocity; SCV, sensory nerve conduction velocity*.

### Correlation of TXNIP With Oxidative Stress and Inflammatory Indexes

#### The Influencing Factors of TXNIP

After multiple stepwise regression taking serum TXNIP as the dependent variable, and age, course of disease, FBG, HblAc, HOMA-IR, vitamin D, SOD, SDBG, TNF-α, GSH, MDA, use of insulin, and use of metformin as the independent variables, TNF-α was identified as a positive influencing factor of TXNIP (*P* < 0.05).

Correlation equation: LogTXNIP = 1.209 + 0.009 TNF-α

#### Influencing Factors for GSH MDA SOD TNF-α in T2DM

After multiple stepwise regression taking serum GSH, MDA, SOD, and TNF-α as dependent variables (due to the non-normal distribution, we used the logarithmically transformed data for GSH and MDA and the square root of the TNF-α data were used in the analysis), and age, course of disease, FBG, HblAc, HOMA-IR, vitamin D, SDBG, TXNIP, use of insulin, and use of metformin as independent variables, serum TXNIP was identified as a positive influencing factor for MDA and TNF-α in T2DM patients (*P* < 0.05).

Correlation equation: LogGSH = 1.384 + 0.012 HOMA-IR

SOD = 45.383 + 0.145 age

LogMDA = 0.899 + 0.001 TXNIP

SqrtTNF-α = 4.079 + 0.180 HblAc – 0.102 FBG + 0.007 TXNIP

## Discussion

TXNIP plays an important role in the process of cell proliferation, differentiation, apoptosis, and the occurrence and development of tumors and stress disorders. Previous studies focused on the relationship between TXNIP and DM, diabetic nephropathy and diabetic retinopathy, while there are no clinical reports on the relationship between serum TXNIP and DPN.

In this study, we investigated the correlation between TXNIP and peripheral NCV in patients with T2DM patients to evaluate the effect of TXNIP on NCV and clarify the effect of TXNIP on DPN these patients. The classification of DPN is related to the involved pattern of peripheral nerve, [i.e., mononeuropathy, multyneuropathy, and orpolyneuropathy (11)]. In this study, we discussed the relationship between TXNIP and NCV abnormality, so we only considered whether there was NCV abnormality in patients. According to NCV of subjects, T2DM patients were divided into NCVN and NCVA groups. Taking abnormal NCV as the dependent variable, and TXNIP, age, course of diabetes, FBG, HbA1c, vitamin D3, and SDBG as independent variables, regression analysis showed that the risk factors for NCV abnormality in T2DM patients were age, course of diabetes, FBG, SDBG, vitamin D, and TXNIP. Age and course of disease were significantly correlated with DPN, which was consistent with the results of previous studies (12). Vitamin D protects the function of peripheral nerves by inhibiting the inflammatory state of DM patients and reducing the apoptosis of neurons (13). In this study, it was found that SDBG, LAGE, and MBG were increased in the NCVA group, and SDBG was an independent risk factor for abnormal NCV; therefore, we concluded that blood glucose fluctuation was related to DPN. Glucose volatility has been shown to be detrimental to endothelial function, inflammatory reactions, and oxidative stress (14). In an animal experiment conducted by Yang et al., blood glucose fluctuations slowed the MCV of the sciatic nerve and damaged the myelin sheath and axon structure of the sciatic nerve via a mechanism related to oxidative stress (15).

After adjusting for other factors, we identified TXNIP as an independent risk factor for NCV abnormality in T2DM patients. At present, the mechanism by which TXNIP contributes to the pathogenesis of NCV abnormality is unclear; however, several explanations can be postulated. First, TXNIP causes NCV abnormality by activating oxidative stress. TXNIP combines with TRX to inhibit the function and expression of TRX (3). TRX, which is ubiquitously expressed in mammalian cells, regulates the REDOX state of cells, removes reactive oxygen species (ROS) *in vivo* and combats oxidative stress. In the cytoplasm, TXNIP binds to TRX to increase ROS levels, ultimately leading to oxidative stress (16). Oxidative stress can damage the neurovascular endothelium, produce toxic effects on neurons and nerve fibers, causing degeneration and necrosis of peripheral nerve tissues, and leading to the occurrence of DPN. In one animal experiment, TXNIP gene expression was increased in the dorsal root ganglia of rats with DM induced by a single intraperitoneal injection of streptozotocin (STZ). One week after the induction of diabetes, the NCV of DM rats was unchanged, whereas TXNIP expression was increased (17), indicating that the upregulation of TXNIP occurred prior to the neurological deficit. This study also showed that the use of antioxidants and p38 protein kinase inhibitors reduced oxidative stress, but did not prevent the increase in TXNIP expression, indicating that oxidative stress is caused by the increase in TXNIP levels. In addition, TXNIP was identified as a positive influencing factor for MDA in T2DM. TXNIP and oxidative stress can influence each other and act together to promote the occurrence and development of DPN.

Second, TXNIP may affect NCV by promoting inflammatory reactions. This hypothesis is supported by the identification of TXNIP as influencing factor on TNF-α levels in the NCVA group. TXNIP plays an important role in the development of inflammation. Binding of TXNIP to TRX induces the release of apoptosis signal regulated kinase 1 (ASK1) and the expression of proinflammatory factors (18). Furthermore, TXNIP upregulation promotes the activate NLRP3 inflammatory corpuscles. NLRP3 is a protein responsible for the regulation of immune inflammation, and its activation can trigger the activation of caspase-1 and the production of inflammatory mediators, ultimately leading to apoptosis of β-cells in the pancreatic islets, leading to insulin resistance (19). The insulin receptor and its substrates are widely expressed in the cell bodies and axons of peripheral neurons. Insulin resistance inhibits the signaling pathways controlling mitochondria, which may have an impact on mitochondrial function in neurons and promote the occurrence and development of DPN (20).

Finally, TXNIP may also affect NCV by causing axonal damage. Axonal injury is a complex process, involving changes in a variety of pathways, including mitochondrial dysfunction, oxidative stress, ischemia, ATP depletion, redistribution of ion channels, axonal transport disorders, and reduced nutritional support (21). Mitochondrial dysfunction is considered as an important step in axonal injury. Inflammation and oxidative stress seriously affect the speed of axon mitochondrial transport. Inflammation preferentially damages retrograde transport, while oxidative stress damages mitochondria more directly than inflammation (22). At the same time, a significant correlation between osteopontin levels and NFL and MBP (factors associated with axonal damage was observed in one study, and NOx was significantly associated with MBP (23). TXNIP may cause axon damage by activating oxidative stress and inflammatory reaction, and slow axonal transport, as well as changes in ion channel dynamics and expression may eventually lead to abnormal nerve conduction velocity (24). The relationship between TXNIP and axon damage needs further study.

In T2DM patients, we found that serum TXNIP was identified as an independent risk factor for left ulnar MCV, right ulnar MCV, left median MCV, and right median MCV, and increased TXNIP levels decreased the NCV in these nerves. It can be concluded that TXNIP is closely related to the MCV of the bilateral upper limbs in this study. The typical manifestation of DPN is a kind of distal symmetrical polyneuropathy (DSPN), which presents a “sock like” distribution in a length-dependent manner. Generally, the lower limbs are more susceptible than the upper limbs. When upper limb symptoms or signs are present, they are more likely to be caused by co-existing single neuropathy than by multiple neuropathy (11). In this study, we found that TXNIP is closely related to the motor conduction velocity of both upper limbs, follow-up studies are required to clarify the specific relationship between TXNIP and differences in NCVs and the underlying mechanisms. Studies are also required to test the hypothesis that TXNIP levels exert selective or preferential effects on motor fibers.

TXNIP expression is affected by a variety of factors. In this study, TXNIP was positively influenced by TNF-α. In addition, TXNIP induces rapid transcriptional regulation, and short-term blood glucose fluctuation can induce TXNIP expression (25). In addition insulin treatment has been shown to normalize TRX activity and inhibit TXNIP levels in animal models. Insulin functions via phosphatidylinositol kinase (PI3K) to degrade TXNIP (26) and metformin reduces TXNIP levels by activating AMPK (27).

Previous animal studies have implicated TXNIP as a therapeutic target for neuroprotection. In pre-DM mice induced by high fat diet, the occurrence of neuropathy is related to the upregulation of TXNIP. Furthermore, when verapamil was used to inhibit TXNIP expression, DPN was improved by inhibiting the activation of inflammatory bodies and reducing neuronal apoptosis (16). In addition, Jinmaitong (JMT) can improve DPN by inhibiting the activation of TXNIP/NLRP3 inflammatory bodies (28). Chen et al. used calcium antagonists, such as verapamil and diltiazem, to reduce the expression of TXNIP, although the mechanism was not clarified (29).

In conclusion, this study shows that elevated serum TXNIP, oxidative stress, and inflammation are present in patients with diabetic neuropathy. TXNIP is an independent risk factor for NCV. TXNIP is closely related to the conduction velocity of motor neurons in both upper limbs. TXNIP may interact with oxidative stress and inflammation to promote the occurrence of DPN, although the exact mechanism requires further exploration.

## Data Availability Statement

All datasets generated for this study are included in the article/Supplementary Material.

## Ethics Statement

This study was approved by the Clinical Research Ethics Committee of Hebei General Hospital (China). The patients/participants provided their written informed consent to participate in this study.

## Author Contributions

YG, SC, MP, and LR designed and planned the study, performed the experiments, and wrote the manuscript. ZW, SM, and MZ performed the experiments and analyzed the data. All authors contributed to the article and approved the submitted version.

## Conflict of Interest

The authors declare that the research was conducted in the absence of any commercial or financial relationships that could be construed as a potential conflict of interest.

## Appendix 1

**Table:**

**Dantec Keypoint normal values**							
Age (years)	15–24	25–34	35–44	45–54	55–64	65–74	75–84
Ulnar MCV (m/s)	61	61	61	61	61	58	52
Media MCV (m/s)	56	55	54	52	51	50	50
Common peroneal MCV (m/s)	43						
Tibial MCV (m/s)	43						
Ulnar SCV (m/s)	47.4	46.6	45.8	45	44.2	43.3	42.9
Media SCV (m/s)	49.5	48.6	47.5	46.5	45.4	44.4	43.4
Superficial peroneal SCV (m/s)	40						
Sural SCV (m/s)	42						

*If the nerve conduction velocity is less than the above indexes, the conduction velocity is abnormal*.
