# Supplementary material for: Correlation Between Thioredoxin-Interacting Protein and Nerve Conduction Velocity in Patients With Type 2 Diabetes Mellitus
Source: Front Neurol. 2020 Jul 22;11:733. doi: 10.3389/fneur.2020.00733 (PMC7387714; doi:10.3389/fneur.2020.00733)
Supplement: Supplementary file 2 [file Data_Sheet_1.PDF]

Hebei General Hospital

Ethics Committee Application for Approval of Research Protocol

NO. 201824

**Paper Title: Correlation between thioredoxin-interacting protein and nerve conduction velocity in patients with type 2 diabetes mellitus**	
**Article author:** Yuan Gao, Shuchun Chen , Minmin Peng , Zi Wang , Luping Ren ,Shumin Mu Meilin Zheng	
**Documents for Application**	■ Application form<br>■the thesis
**Consequence**	**After reviewing ,this paper conforms to the principals of medical ethics .It is accepted to publish.**

河北省人民医院 医学伦理委员会

Committee Leader（Signature）:Yanhui Peng

Hebei General Hospital Ethics Committee

Date: 2018.12.26

Adress:NO.348 Heping West Road Shijiazhuang City, Hebei Prov. CHINA
TEL:0311–85988311
